# Prognostic implications of systemic immune-inflammation index in myocardial infarction patients with and without diabetes: insights from the NOAFCAMI-SH registry

**DOI:** 10.1186/s12933-024-02129-x

**Published:** 2024-01-22

**Authors:** Jiachen Luo, Xiaoming Qin, Xingxu Zhang, Yiwei Zhang, Fang Yuan, Wentao Shi, Baoxin Liu, Yidong Wei, the NOAFCAMI-SH Registry Investigators

**Affiliations:** Department of Cardiology, Shanghai Tenth People’s Hospital, Tongji University School of Medicine, 301 Middle Yanchang Road, Jingan District, Shanghai, 200072 China

**Keywords:** Myocardial infarction, Diabetes, Systemic immune-inflammation index, Generalized additive model, Restricted cubic spline

## Abstract

**Background:**

It is well-known that systemic inflammation plays a crucial role in the pathogenesis and prognosis of acute myocardial infarction (AMI). The systemic immune-inflammation index (SII, platelet × neutrophil/lymphocyte ratio) is a novel index that is used for the characterization of the severity of systemic inflammation. Recent studies have identified the high SII level as an independent predictor of poor outcomes in patients with AMI. We aimed to investigate the prognostic implications of SII in AMI patients with and without diabetes mellitus (DM).

**Methods:**

We included 2111 patients with AMI from February 2014 to March 2018. Multivariable Cox regression analyses were performed to estimate the hazard ratios (HRs) and 95% confidence intervals (CIs) of all-cause death and cardiovascular (CV) death. Multiple imputation was used for missing covariates.

**Results:**

Of 2111 patients (mean age: 65.2 ± 12.2 years, 77.5% were males) analyzed, 789 (37.4%) had DM. Generalized additive model analyses showed that as the SII increased, the C-reactive protein and peak TnT elevated while the LVEF declined, and these associations were similar in patients with and without DM. During a median of 2.5 years of follow-up, 210 all-cause deaths and 154 CV deaths occurred. When treating the SII as a continuous variable, a higher log-transformed SII was significantly associated with increased all-cause mortality (HR: 1.57, 95%CI: 1.02–2.43) and CV mortality (HR: 1.85, 95%CI 1.12–3.05), and such an association was also significant in the diabetics (HRs and 95%CIs for all-cause death and CV death were 2.90 [1.40–6.01] and 3.28 [1.43–7.57], respectively) while not significant in the nondiabetics (P_interaction_ for all-cause death and CV death were 0.019 and 0.049, respectively). Additionally, compared to patients with the lowest tertiles of SII, those with the highest tertiles of SII possessed significantly higher all-cause mortality (HR: 1.82, 95%CI 1.19–2.79) and CV mortality (HR: 1.82, 95%CI 1.19–2.79) after multivariable adjustment, and this relationship remained pronounced in the diabetics (HRs and 95%CIs for all-cause death and CV death were 2.00 [1.13–3.55] and 2.09 [1.10–3.98], respectively) but was not observed in the nondiabetics (HRs and 95%CIs for all-cause death and CV death were 1.21 [0.75–1.97] and 1.60 [0.89–2.90], respectively). Our restricted cubic splines analyses indicated a pronounced linear association between SII and mortality only in diabetics.

**Conclusions:**

In AMI patients with DM, high SII is an independent predictor of poor survival and may be helpful for patient’s risk stratification.

**Supplementary Information:**

The online version contains supplementary material available at 10.1186/s12933-024-02129-x.

## Introduction

Acute myocardial infarction (AMI) is one of the leading causes of death worldwide and accounts for more than 1/3 of all deaths in developed countries [1]. Evolving evidence has suggested that the blood cells such as macrophages, neutrophils, monocytes, and platelets are involved in systemic inflammation and are associated with the pathogenesis of atheroprogression and plaque invulnerability [2, 3]. Upon activation, immune cells will produce and secrete a large number of proinflammatory cytokines including interleukin-8 (IL-8), IL-6, and IL-1β, which have been linked to the outcomes of AMI patients [4–6]. Nevertheless, previous research exploring the clinical usefulness of anti-inflammatory therapies in AMI has obtained controversial results [7], some studies support the utility of anti-inflammatory drugs [8, 9], while others are against it [10, 11]. It is possible that the application of adequate inflammatory biomarkers for the post-MI systemic inflammation evaluation may be helpful in the decision-making of anti-inflammatory treatment.

Until now, the predictive performance of several inflammatory biomarkers, for example, the high-sensitivity C-reactive protein (hs-CRP) and IL-6, has been studied in AMI individuals [12]. More recently, a novel index, the systemic immune-inflammation index (SII), is proposed by Hu et al. to facilitate the risk stratification of patients with hepatocellular carcinoma. The SII is calculated using neutrophil, lymphocyte, and platelet counts (SII = platelet count × neutrophil/lymphocyte ratio), which considers an individual’s inflammatory and immune status simultaneously [13]. Since then, it has been mentioned that SII may be associated with poor outcomes in patients with various cardiovascular diseases [14, 15].

Diabetes mellitus (DM) and AMI are highly prevalent, aggravate each other, and own shared risk factors, of which inflammation plays a crucial role in the pathogenesis and prognosis of both conditions [16, 17]. Recent studies have reported that in patients with DM, the CV benefits of antidiabetic agents are not solely dependent on their hypoglycemic effects, but are also partially due to their anti-inflammation properties [18, 19]. Although the prognostic value of SII has been studied in patients with AMI, the association between SII and clinical outcomes in AMI patients with DM remains unclear.

Accordingly, using data from the New-Onset Atrial Fibrillation Complicating Acute Myocardial Infarction in ShangHai (NOAFCAMI-SH) registry, we intend to investigate the prognostic implications of SII in long-term mortality in AMI patients with and without DM.

## Materials and methods

### Study design and population

This investigation is a retrospective sub-analysis of the NOAFCAMI-SH registry (ClinicalTrials.gov, NCT03533543). The detailed study designs have been previously reported elsewhere [20, 21]. In brief, a total of 2399 patients with AMI who did not have a medical record of AF and were hospitalized in the coronary care unit of Shanghai Tenth People’s Hospital between February 2014 and March 2018 were included in the NOAFCAMI-SH registry. Information about patients’ demographics, concomitant diseases, admission characteristics, laboratory tests, echocardiographic and angiographic data, as well as medications was comprehensively collected and stored in an electronic database.

**Fig. 1:**
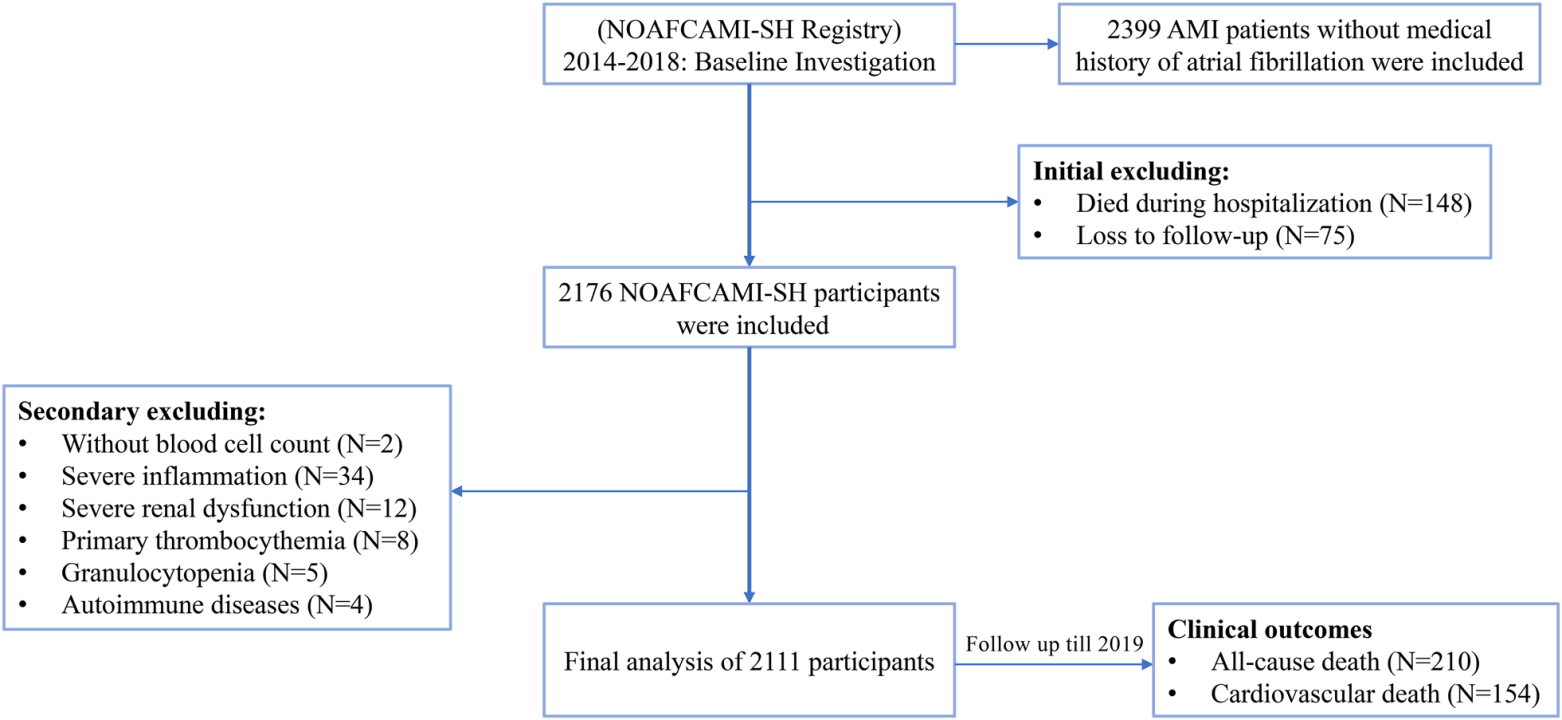
Flowchart of patient inclusion and exclusion for this study. AMI, acute myocardial infarction

For the present analysis, patients meeting the following criteria were excluded: (1) patients who died during hospitalization or lost to follow-up, (2) severe inflammation (leukocyte counts ≥ 20 × 10^9^ cells/L or C-reactive protein ≥ 200 mg/L), (3) severe chronic kidney disease (CKD) with an estimated glomerular filtration rate < 15 mL/min/1.73 m^2^, (4) medical history of hematological diseases (thrombocythemia and granulocytopenia) and autoimmune diseases. Hence, 2111 AMI patients with complete follow-up were included in the final analysis (Fig. 1). The study process was in accordance with the Declaration of Helsinki and was approved by Shanghai Tenth People’s Hospital Ethics Review Committee (approval number: SHSY-IEC-KY-4.1/18–199/01). Due to the anonymous nature of the data, the requirement for informed consent was waived.

### Data collection

The patient’s baseline characteristics, which included demographics, comorbidities, laboratory data, medication usage, and echocardiographic and angiographic information were retrieved from the NOAFCAMI-SH registry database. Demographics included age, sex, and smoking status. Comorbidities included hypertension, diabetes, hyperlipidemia, chronic kidney disease (CKD), heart failure (HF), myocardial infarction (MI) percutaneous coronary intervention (PCI), and stroke/transient ischemic attack (TIA). Venous blood samples were obtained from the cubital vein within 24 h after the index AMI admission. Laboratory data included complete blood count, C-reactive protein (CRP), serum creatinine, and peak level of TnT. The complete blood count including leukocyte, neutrophil, lymphocyte, and platelet counts was examined using the CELL-DYN 3700 (Abbott Laboratories, Illinois, United States) in a central laboratory. An echocardiogram examination was performed by sonologists within 7 days after admission to evaluate the following parameters: left ventricular ejection fraction (LVEF), left atrial diameter, left ventricular end-diastolic diameter (LVEDD), and left ventricular end-systolic diameter (LVESD). Medications included the use of aspirin, angiotensin-converting enzyme inhibitor/angiotensin receptor blocker/ angiotensin receptor–neprilysin inhibitor (ACEI/ARB/ARNI), β-blocker, and statin. All data were collected by trained independent investigators.

### Definitions

The diagnosis of AMI was established based on the contemporary guidelines as an ST-segment elevation MI (STEMI) or non-ST-segment elevation MI (NSTEMI) [22]. DM was defined as a self-reported history of DM, or patients with fasting glucose > 126 mg/dL or HbA1c > 6.5%, or those taking insulin or other antidiabetic treatments. Hypertension was defined as a self-reported history of hypertension, an SBP ≥ 140 mmHg and/or DBP ≥ 90 mmHg, or the use of antihypertensive medications. CKD was defined as patients with an eGFR < 60 ml/min/1.73 m^2^, which was estimated using the Chronic Kidney Disease Epidemiology Collaboration equation. The SII was defined as follows: total peripheral platelets count × neutrophil-to-lymphocyte ratio/10^9^ cells/L. The analyzed population was divided into three groups according to the tertiles of SII (Tertile 1: SII < 613.57; Tertile 2: 613.57 ≤ SII < 1136.56; Tertile 3: SII ≥ 1136.56).

### Outcomes and follow-up

The primary outcome was all-cause death and the secondary outcome was cardiovascular death (CV death). All deaths without a validated non-cardiovascular cause would be considered CV death. All patients were followed from the index discharge to the date of occurrence of an outcome of interest, or last follow-up (April 2019), whichever came first. Hence, the adjudication of clinical outcomes cannot be influenced by the COVID-19 pandemic. For patients who died during hospitalization, mortality data were obtained from medical records. For those dying in the community, mortality data were collected by telephone interviews with the next-of-kin. Survival status was adjudicated by trained physicians who were blinded to the patient’s clinical and laboratory data.

### Statistical analysis

We used means ± SD to present continuous variables with a normal distribution and median (interquartile range) for those with a skewed distribution. The ANOVA and rank sum tests were used to explore differences in measurement data with normal and skewed distribution between the SII tertiles, respectively. Categorical variables were presented by SII tertiles and percentages. We used Fisher’s exact test for dichotomous variables.

A generalized additive model (GAM) and smoothing curve fitting were used to assess the potential relationship between log-transformed SII and inflammation and myocardial injury indicators (CRP, peak TnT, and LVEF levels). SII truncation was performed on all patients outside the 99th percentile to avoid the effect of extreme values.

For long-term survival analyses, the cumulative mortality was plotted using the Kaplan–Meier method, and the differences across three groups were compared using the log-rank test. The incidence rate was calculated using the total number of deaths during the observational period divided by person-years at risk. The association of SII (either as a continuous variable [transformed to a logarithmic scale] or a categorical variable treating the lowest tertiles of SII as reference) with mortality was evaluated using the multivariable Cox proportional hazards regression analysis in the whole, diabetic, and non-diabetic cohorts, respectively. Covariates avoiding collinearity in multivariable models were selected based on the P-value (< 0.05) in univariable analysis (Additional file 1: Table S1) or clinical plausibility, including age, sex, current smoker, comorbidities (hypertension, diabetes, dyslipidemia, CKD, HF, and MI), STEMI, Killip > I, primary PCI, peak TnT, CRP, serum creatinine, LVEF, as well as medications (aspirin, ACEI/ARB/ARNI, β-blocker). Multiple imputation via chained equations was used to impute missing values in CRP (N = 102, 4.8%), serum creatinine (N = 26, 1.2%), and LVEF (N = 97, 4.6%). We assumed that data were missing at random and modeled missing data with the predictive mean matching method. Pooled analyses across the 20 imputed datasets followed Rubin’s rules (Additional file 1: Methods) [23]. To investigate the single impacts of leukocyte subtypes on patient’s prognosis, we further explored whether there were associations between lymphocyte, neutrophil, platelet counts, and mortality. We also performed three sensitivity analyses to examine the robustness of our findings: (1) repeating the multivariable analyses in a complete-case dataset by removing covariates with missing data; and further accounting for the impacts of (2) oral anticoagulants and diuretics, as well as (3) antidiabetic agents in the multivariable models.

The potential nonlinear correlations between SII level and long-term mortality were assessed on a continuous scale with the restricted cubic spline (RCS) method. Three equally spaced knots were set at 10th, 50th, and 90th percentiles. The hazard ratios (HR) and 95% confidence intervals (CI) were calculated after adjustment for the aforementioned covariates. We conducted all statistical analyses using R v4.0.3 software (‘mice’, ‘mgcv’, ‘rms’ packages) and Stata v17.0 software. A two-sided p-value < 0.05 was considered statistically significant.

## Results

The baseline characteristics divided by SII tertiles were displayed in Table 1. Among the analyzed participants, 1637 (77.5%) were males; the mean age was 65.2 years (SD: 12.2 years). Pronounced differences in the medical history of CKD and PCI, admission characteristics (STEMI, heart rate, and Killip class), laboratory indices (CRP, peak TnT, creatinine, as well as leukocyte, neutrophil, lymphocyte, and platelet counts), usage of primary PCI and medications (aspirin, ACEI/ARB/ARNI, β-blocker, diuretics, and insulin), and LVEF level were observed across the SII tertiles. Additional file 1: Tables S2 and S3 summarized the baseline characteristics of AMI patients with and without DM, respectively. The characteristics including CRP, peak TnT, complete blood counts, and LVEF level remained significantly different between SII tertiles in both cohorts.

**Table 1:** Baseline characteristics of AMI patients by tertiles of systemic-immune inflammation index

Clinical characteristics	Overall (N = 2111)	Tertiles of systemic-immune inflammation index			
		Tertile 1 (N = 702)	Tertile 2 (N = 706)	Tertile 3 (N = 703)	P-value
		< 613.57	613.57–1136.56	≥ 1136.56	
Age, years	65.2 ± 12.2	65.4 ± 12.0	64.6 ± 12.4	65.5 ± 12.2	0.339
Male sex	1637 (77.5)	534 (76.1)	552 (78.2)	551 (78.4)	0.515
Current smoker	953 (45.1)	303 (43.2)	343 (48.6)	307 (43.7)	0.078
Comorbidities					
Hypertension	1342 (63.6)	441 (62.8)	444 (62.9)	457 (65.0)	0.628
Diabetes mellitus	789 (37.4)	277 (39.5)	258 (36.5)	254 (36.1)	0.377
Dyslipidemia	565 (26.8)	183 (26.1)	187 (26.5)	195 (27.7)	0.763
Chronic kidney disease	165 (7.8)	40 (5.7)	61 (8.6)	64 (9.1)	0.036
History of HF	105 (5.0)	28 (4.0)	33 (4.7)	44 (6.3)	0.133
History of MI	137 (6.5)	57 (8.1)	41 (5.8)	39 (5.6)	0.098
History of PCI	187 (8.9)	84 (12.0)	49 (6.9)	54 (7.7)	0.002
History of stroke/TIA	236 (11.2)	64 (9.1)	87 (12.3)	85 (12.1)	0.104
Admission presentation					
STEMI	1272 (60.3)	327 (46.6)	439 (62.2)	506 (72.0)	< 0.001
Anterior location^a^	654 (51.4)	155 (47.4)	234 (53.3)	265 (52.4)	0.401
Systolic BP, mmHg	138.5 ± 23.9	139.2 ± 22.7	139.2 ± 23.4	136.9 ± 25.4	0.123
Heart rate, bpm	79.6 ± 16.7	76.3 ± 15.7	79.6 ± 15.4	83.0 ± 18.2	< 0.001
Killip class > I	279 (13.2)	68 (9.7)	76 (10.8)	135 (19.2)	< 0.001
Laboratory indices					
C-reactive protein, mg/L	5.38 (3.17–17.20)	3.97 (3.02–12.80)	5.69 (3.27–17.26)	6.86 (3.17–25.90)	< 0.001
Peak TnT, ng/mL	2.97 (0.78–7.98)	1.55 (0.43–4.88)	2.90 (0.97–7.27)	5.36 (1.63–10.00)	< 0.001
Serum creatinine, mg/dL	0.95 ± 0.33	0.93 ± 0.31	0.95 ± 0.34	0.98 ± 0.33	0.018
Leukocyte count, 10^9^/L	9.54 ± 3.04	7.78 ± 2.08	9.30 ± 2.54	11.60 ± 3.13	< 0.001
Neutrophil, count 10^9^/L	7.14 ± 2.92	4.88 ± 1.45	6.88 ± 2.00	9.66 ± 2.82	< 0.001
Lymphocyte count, 10^9^/L	1.72 ± 0.79	2.22 ± 0.88	1.72 ± 0.62	1.22 ± 0.50	< 0.001
Platelet count, 10^9^/L	207.1 ± 59.6	181.5 ± 48.7	206.8 ± 54.8	233.1 ± 63.0	< 0.001
Log-transformed SII	2.93 ± 0.31	2.60 ± 0.15	2.91 ± 0.07	3.27 ± 0.18	< 0.001
Angiographic data					
Primary PCI	1778 (84.2)	567 (80.8)	609 (86.3)	602 (85.6)	0.008
Infarct-related artery^b^					0.075
Left anterior descending	626 (51.6)	146 (47.1)	232 (54.7)	248 (51.8)	
Right coronary artery	455 (37.5)	127 (41.0)	140 (33.0)	188 (39.2)	
Left circumflex	132 (10.9)	37 (11.9)	52 (12.3)	43 (9.0)	
Echocardiographic data					
LAD, mm	38.1 ± 4.6	38.3 ± 4.4	38.2 ± 4.7	37.8 ± 4.7	0.171
LVESD, mm	31.0 ± 5.5	30.7 ± 5.4	30.7 ± 5.3	31.5 ± 5.6	0.015
LVEDD, mm	45.6 ± 4.8	45.7 ± 4.8	45.5 ± 4.6	45.6 ± 4.9	0.889
LVEF, %	50.5 ± 10.5	52.7 ± 9.8	51.1 ± 10.2	47.7 ± 11.0	< 0.001
Medication at discharge					
Aspirin	1946 (92.2)	633 (90.2)	662 (93.8)	651 (92.6)	0.037
ACEI/ARB/ARNI	1262 (59.8)	445 (63.4)	429 (60.8)	388 (55.2)	0.006
β-blocker	1528 (72.4)	485 (69.1)	511 (72.4)	532 (75.7)	0.022
Statin	2026 (96.0)	677 (96.4)	679 (96.2)	670 (95.3)	0.528
Oral anticoagulants	12 (0.6)	6 (0.9)	2 (0.3)	4 (0.6)	0.291
Diuretics	302 (14.3)	79 (11.3)	102 (14.5)	121 (17.2)	0.006
Antidiabetic agents	636 (30.1)	220 (31.3)	205 (29.0)	211 (30.0)	0.642
Insulin	255 (12.1)	68 (9.7)	84 (11.9)	103 (14.7)	0.017
Metformin	231 (10.9)	88 (12.5)	66 (9.4)	77 (11.0)	0.161
Sulfonylureas	213 (10.1)	83 (11.8)	71 (10.1)	59 (8.4)	0.104
Glinides	41 (1.9)	17 (2.4)	11 (1.6)	13 (1.9)	0.490
Thiazolidinediones	19 (0.9)	9 (1.3)	6 (0.9)	4 (0.6)	0.354

^a^For patients with STEMI

^b^For patients with STEMI undergoing angiography

*AMI* acute myocardial infarction, *ACEI* angiotensin-converting enzyme inhibitor, *ARB* angiotensin receptor blocker, *ARNI* angiotensin receptor-neprilysin inhibitor, *BP* blood pressure, *GRACE* Global Registry of Acute Coronary Events, *HF* heart failure, *LAD* left atrial diameter, *LVEDD* left ventricular end-diastolic diameter, *LVEF* left ventricular ejection fraction, *LVESD* left ventricular end-systolic diameter, *PCI* percutaneous coronary intervention, *SII* systemic-immune inflammation index, *STEMI* ST-segment elevation myocardial infarction, *TIA* transient ischemic attack

**Fig. 2:**
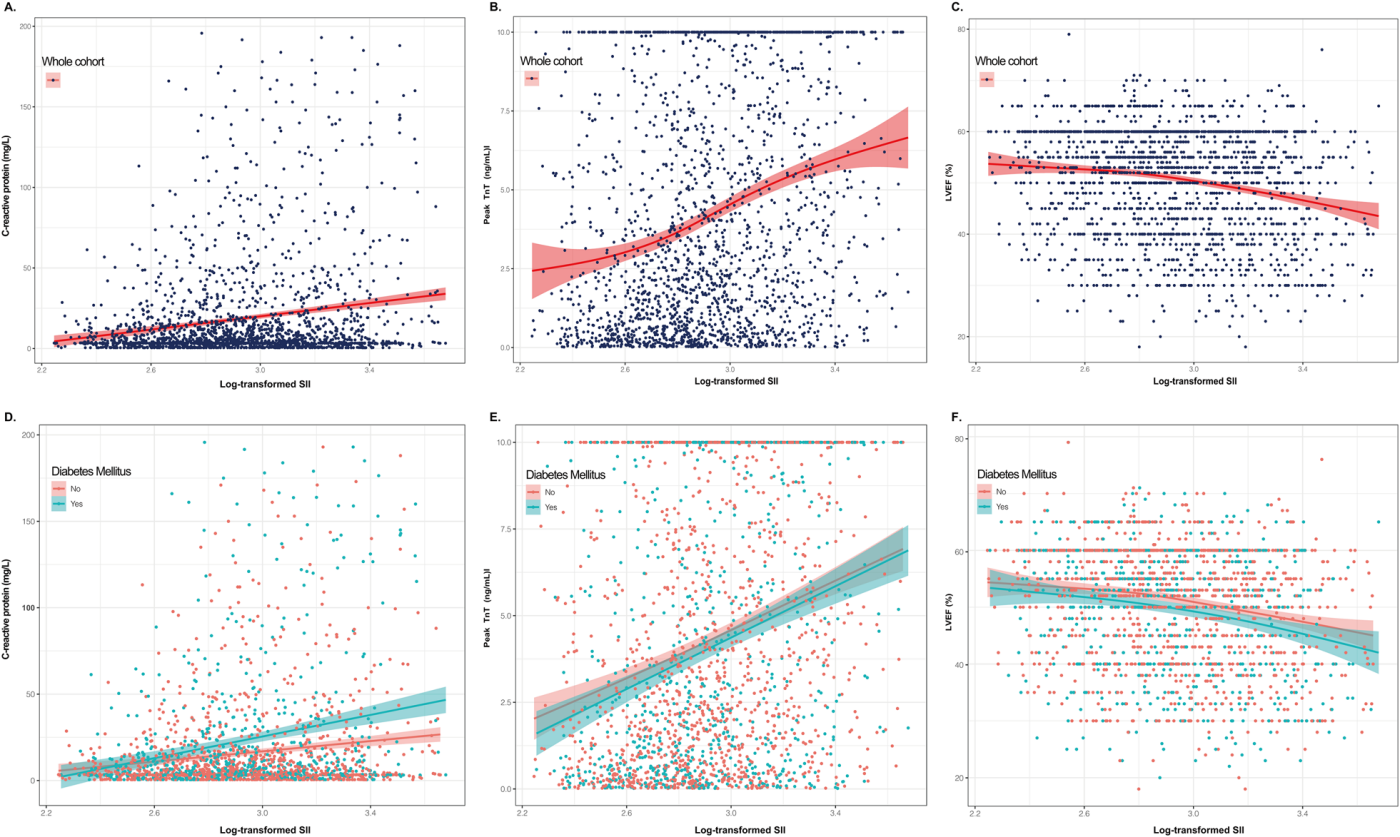
Associations of systemic immune-inflammation index (SII) with inflammation, myocardial injury, and cardiac function assessed with GAM. The generalized additive models (GAM) show a nearly linear association between the log-transformed SII level and CRP, peak TnT, and LVEF levels in the overall cohort, and in the diabetic and nondiabetic cohorts, respectively. The 95% confidence intervals are in shading. The navyblue dots indicate the whole cohort, and the green and red dots indicate the diabetic and nondiabetic cohorts, respectively. *CRP* C-reactive protein, *LVEF* left ventricular ejection fraction

The GAM model was applied to explore the associations between SII and CRP, peak TnT, and LVEF levels. The estimated smooth effect curves were shown in Fig. 2. As log-transformed SII increased, the CRP and peak TnT levels elevated while the LVEF level declined (Figs. 2A–C). Qualitatively similar visual patterns were observed for the diabetic and non-diabetic cohorts (Figs. 2D-F). Notably, the association between log-transformed SII and CRP appeared to be stronger among individuals with DM than among those without DM.

**Fig. 3:**
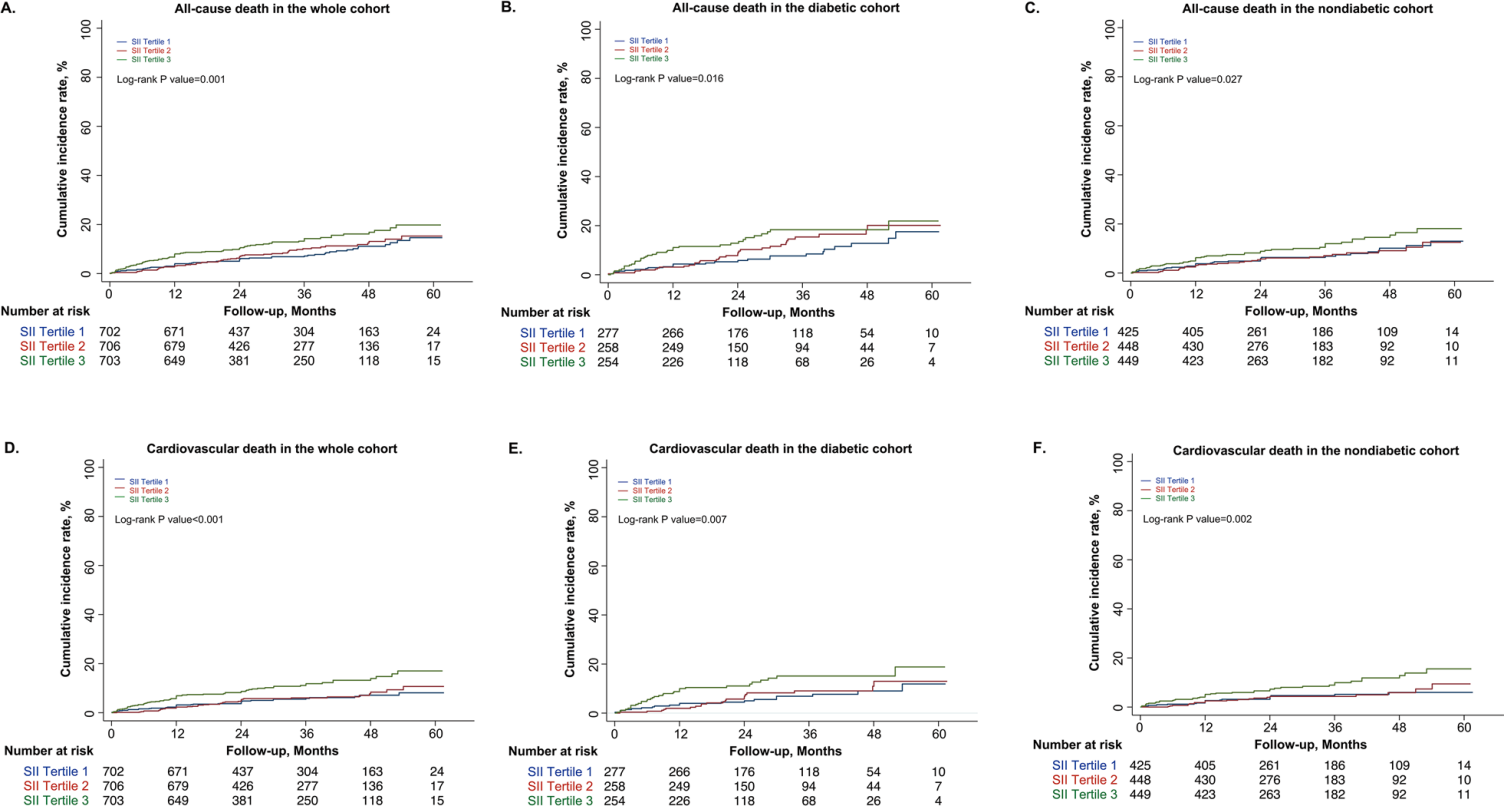
Primary and secondary endpoints. Long-term all-cause mortality and CV mortality in the whole cohort (**A**, **D**), and in the diabetic (**B**, **E**), and nondiabetic (**C**, **F**) cohorts according to the tertiles of SII level, respectively. *CV* cardiovascular

Over a median of 2.5 years (maximum 5.1 years) of follow-up, 210 all-cause deaths and 154 CV deaths were identified. Cumulative hazard curves of all-cause death and CV death across SII tertiles were illustrated in Fig. 3. Patients in the SII tertile 3 group suffered higher all-cause mortality and CV mortality compared to those in the SII tertiles 1 and 2 groups (all p values of log-rank test < 0.05). In the fully adjusted Cox regression models, when treating the lowest tertiles of SII as the reference, the highest tertiles of SII demonstrated significantly positive associations with all-cause death (HR: 1.54, 95%CI 1.07–2.21, P for trend = 0.016) and CV death (HR: 1.82, 95%CI 1.19–2.79, P for trend = 0.004) among the whole population, especially for those with DM (HRs and 95%CIs for all-cause death and CV death were 2.00 [1.13–3.55] and 2.09 [1.10–3.98], respectively), whereas an unpronounced result was obtained in the non-diabetic cohort (HRs and 95%CIs for all-cause death and CV death were 1.21 [0.75–1.97] and 1.60 [0.89–2.90], respectively). Similar trends were found when introducing the SII as a continuous variable into the multivariable models (Table 2). On average, the HR was increased by 57% (HR: 1.57, 95%CI 1.02–2.43) and 1.9-fold (HR: 2.90, 95%CI 1.40–6.01) for all-cause death; and by 85% (HR: 1.85, 95%CI 1.12–3.05) and 2.28-fold (HR: 3.28, 95%CI 1.43–7.57) for CV death in the whole and diabetic cohorts, respectively. When repeating the main analysis in individuals without missing data, sensitivity analyses showed slightly elevated correlations between SII and all-cause death and CV death (Additional file 1: Table S4). Moreover, when further accounting for the impacts of oral anticoagulants and diuretics (Additional file 1: Table S5), as well as the antidiabetic agents (Additional file 1: Table S6) on the association between SII and long-term mortality, the results of which were similar to that in the main analysis.

**Table 2:** Association between tertiles of systemic immune-inflammation index and death in patients with and without diabetes

Clinical outcomes	Log-transformed SII	Tertiles of systemic immune-inflammation index			P for trend
		Tertile 1 (N = 702)	Tertile 2 (N = 706)	Tertile 3 (N = 703)	
		< 613.57	613.57–1136.56	≥ 1136.56	
All-cause death					
Overall cohort (N = 2111)					
Event	–	57	63	90	–
Incidence rate (95%CI)^a^	–	2.95 (2.28–3.83)	3.35 (2.62–4.29)	5.23 (4.25–6.43)	–
Unadjusted Model	2.83 (1.83–4.38)**	Ref.	1.14 (0.80–1.63)	1.75 (1.26–2.44)**	< 0.001
Adjusted Model 1^b^	2.16 (1.46–3.20)**	Ref.	1.18 (0.83–1.69)	1.74 (1.25–2.43)*	< 0.001
Adjusted Model 2^c^	1.57 (1.02–2.43)*	Ref.	1.04 (0.72–1.50)	1.54 (1.07–2.21)*	**0.016**
Diabetic cohort (N = 789)					
Event	–	25	32	39	–
Incidence rate (95%CI)^a^	–	3.32 (2.24–4.91)	4.75 (3.36–6.72)	6.97 (5.09–9.54)	–
Unadjusted Model	4.47 (2.28–8.78)**	Ref.	1.43 (0.85–2.41)	2.05 (1.24–3.40)*	0.005
Adjusted Model 1^b^	3.86 (1.99–7.48)**	Ref.	1.39 (0.82–2.35)	2.08 (1.25–3.44)*	0.004
Adjusted Model 2^c^	2.90 (1.40–6.01)*	Ref.	1.28 (0.74–2.21)	2.00 (1.13–3.55)*	**0.020**
Nondiabetic cohort (N = 1322)					
Event	–	32	31	51	–
Incidence rate (95%CI)^a^	–	2.72 (1.92–3.84)	2.57 (1.81–3.66)	4.39 (3.34–5.77)	–
Unadjusted Model	2.23 (1.24–4.01)*	Ref.	0.95 (0.58–1.56)	1.61 (1.03–2.51)*	0.026
Adjusted Model 1^b^	1.64 (0.98–2.74)	Ref.	1.04 (0.97–1.70)	1.57 (1.01–2.45)*	0.038
Adjusted Model 2^c^	0.97 (0.56–1.69)	Ref.	0.91 (0.54–1.53)	1.21 (0.75–1.97)	0.386
Cardiovascular death					
Overall cohort (N = 2111)					
Event	–	38	41	75	–
Incidence rate (95%CI)^a^	–	1.97 (1.43–2.70)	2.18 (1.61–2.96)	4.36 (3.47–5.46)	–
Unadjusted Model	3.88 (2.35–6.39)**	Ref.	1.10 (0.71–1.72)	2.17 (1.46–3.20)**	< 0.001
Adjusted Model 1^b^	2.90 (1.84–4.57)**	Ref.	1.15 (0.74–1.79)	2.16 (1.46–3.20)**	< 0.001
Adjusted Model 2^c^	1.85 (1.12–3.05)*	Ref.	1.01 (0.64–1.60)	1.82 (1.19–2.79)*	**0.004**
Diabetic cohort (N = 789)					
Event	–	19	20	33	–
Incidence rate (95%CI)^a^	–	2.52 (1.61–3.95)	2.97 (1.92–4.60)	5.90 (4.19–8.29)	–
Unadjusted Model	5.61 (2.58–12.17)**	Ref.	1.16 (0.62–2.18)	2.23 (1.27–3.93)*	0.004
Adjusted Model 1^b^	4.92 (2.30–10.51)**	Ref.	1.15 (0.61–2.15)	2.26 (1.28–3.99)*	0.004
Adjusted Model 2^c^	3.28 (1.43–7.57)*	Ref.	1.09 (0.56–2.09)	2.09 (1.10–3.98)*	**0.026**
Nondiabetic cohort (N = 1322)					
Event	–	19	21	42	–
Incidence rate (95%CI)^a^	–	1.61 (1.03–2.53)	1.74 (1.14–2.67)	3.61 (2.67–4.89)	–
Unadjusted Model	3.25 (1.65–6.41)**	Ref.	1.08 (0.58–2.01)	2.22 (1.29–3.81)*	0.002
Adjusted Model 1^b^	2.24 (1.24–4.07)*	Ref.	1.17 (0.63–2.19)	2.17 (1.26–3.74)*	0.003
Adjusted Model 2^c^	1.14 (0.60–2.16)	Ref.	1.06 (0.55–2.02)	1.60 (0.89–2.90)	0.097

*P < 0.05; **P < 0.001

^a^Incidence rate was calculated using the total number of deaths during the observational period divided by person-years at risk

^b^Model 1 included age and sex

^c^Model 2 included age, sex, current smoker, comorbidities (hypertension, diabetes, dyslipidemia, CKD, HF, and MI), STEMI, Killip > I, primary PCI, peak TnT, CRP, serum creatinine, LVEF, as well as medications (aspirin, ACEI/ARB/ARNI, β-blocker)

*CKD* chronic kidney disease, *CRP* C-reactive protein, *HF* heart failure, *LVEF* left ventricular ejection fraction, *PCI* percutaneous coronary intervention, *SII* systemic immune-inflammation index

The bold value represents the statistical significance of 'P for trend' (P for trend<0.05) in the fully-adjusted model

**Fig. 4:**
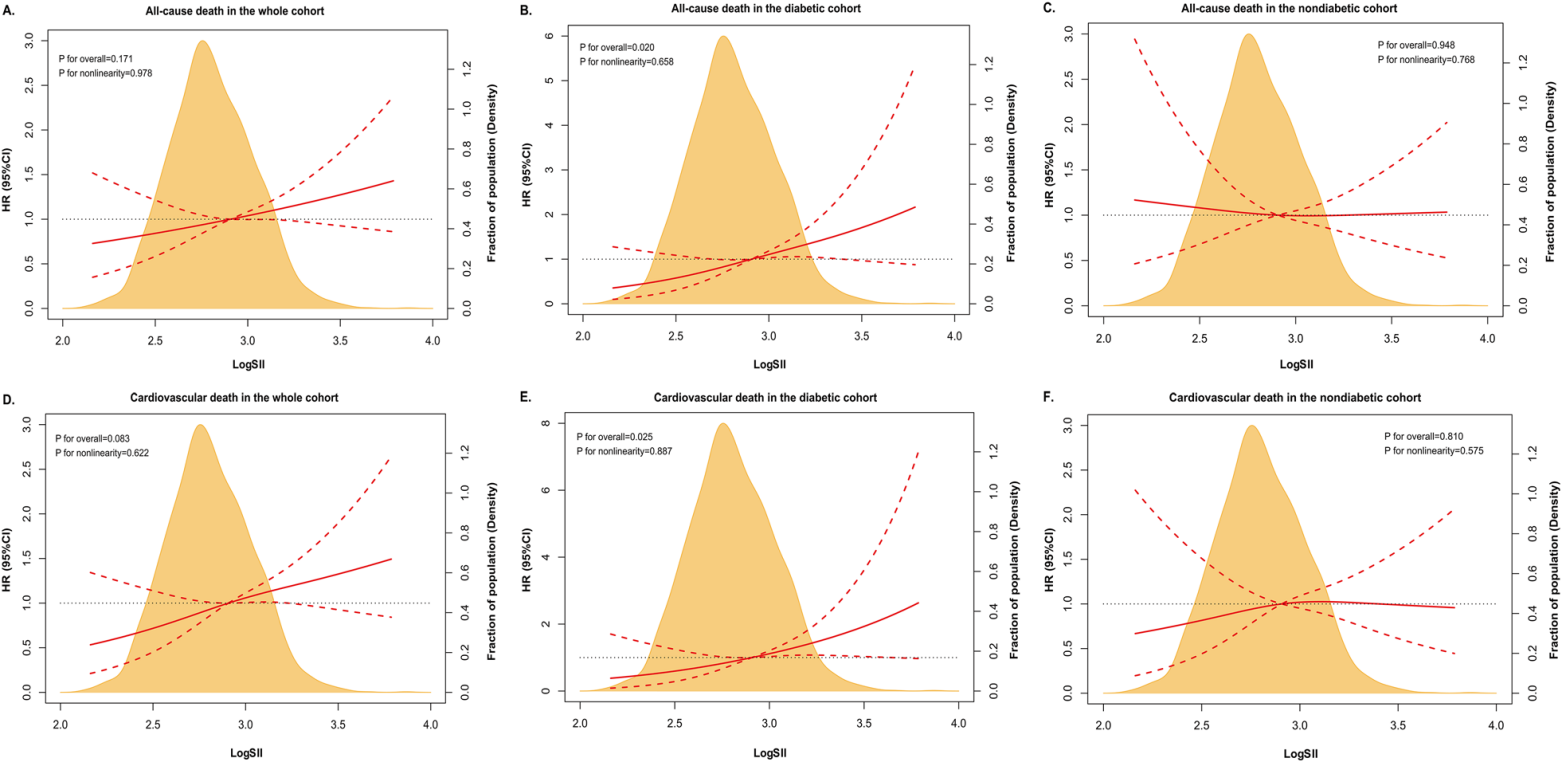
Hazard ratios (HRs) for all-cause death and CV death by SII on a continuous scale in the whole cohort, and in the diabetic and nondiabetic cohorts. HRs with 95%CIs for all-cause death and CV death, accounting for SII on a continuous scale, are from Cox regression restricted cubic splines (RCS). Orange areas show the distribution of SII levels. The solid red line in each figure indicates the HR, and the dashed red lines indicate the 95%CI. Covariates in the multivariable RCS model included: age, sex, current smoker, comorbidities (hypertension, diabetes, dyslipidemia, CKD, heart failure, myocardial infarction), STEMI, Killip > I, primary PCI, peak TnT, CRP, serum creatinine, LVEF, as well as medications (aspirin, ACEI/ARB/ARNI, β-blocker). *CKD* chronic kidney disease, *PCI* percutaneous coronary intervention

As shown in Fig. 4, dose–response relationships between log-transformed SII and all-cause death and CV death were plotted using RCS. Significant linear associations between SII and all-cause mortality (P_overall_ = 0.020, P_nonlinearity_ = 0.658) and CV mortality (P_overall_ = 0.025, P_nonlinearity_ = 0.887) were only found in the diabetic cohort (Fig. 4B, E), while no evident associations between SII and death were observed in patients with DM (all P_overall_ > 0.05).

Furthermore, when investigating the associations of leukocyte subtypes with death, we demonstrated that higher log-transformed neutrophil counts were significantly associated with increased all-cause mortality (HR: 4.59, 95%CI 1.26–16.70, P = 0.024) and CV mortality (HR: 5.37, 95%CI 1.22–23.68, P = 0.031) in the diabetic cohort after accounting for confounders (Additional file 1: Table S7). A greater log-transformed platelet count was independently associated with increased all-cause mortality (HR: 3.24, 95%CI 1.16–9.02, P = 0.026) in the whole population and a marginally elevated mortality was observed in the diabetics (HR: 4.36, 95%CI 0.94–20.15, P = 0.063) (Additional file 1: Table S8). However, lymphocyte counts were not associated with poor survival (Additional file 1: Table S9).

## Discussions

The principal findings of this study were as follows: (1) there was a positive association between SII level and myocardial injury and cardiac dysfunction after AMI; (2) patients with a greater SII level experienced higher risk of all-cause and CV deaths compared to those with lower SII; (3) high SII remained an independent predictor of long-term mortality in the diabetic cohort after multivariable adjustment, and a linear association between SII and poor survival was further uncovered in the RCS analyses.

Preceding studies have identified chronic inflammation as an important risk factor for several diseases such as cancer, DM, and atherosclerotic disease [24]. The SII was a novel biomarker that was used for the characterization of systemic inflammation, which is evaluated using neutrophil, lymphocyte, and platelet counts [13]. It is well-known that the immune cells are involved in cardiac injury and repair [25], and numerous studies have suggested the SII as an independent predictor of adverse CV outcomes after AMI, for example, mortality, arrhythmia, and stent thrombosis [26–28]. From the mechanism perspective, activated neutrophils release a variety of proteolytic enzymes such as myeloperoxidase and elastase, thus leading to myocardial injury [29]. By contrast, lymphocytes represent a regulated inflammatory process that suppresses the exorbitant immune response and limits myocardial damage. Upon activation, platelets will either release a number of proinflammatory chemokines and cytokines that contribute to thrombosis or interact with other leukocytes to exacerbate atherosclerosis and plaque instability which is often related to detrimental CV outcomes [30].

In a retrospective cohort study of 314 elderly patients with NSTEMI, Orhan et al. demonstrated that a greater level of SII was significantly associated with increased in-hospital mortality and long-term mortality after adjusting for age, DM, hypertension, HF, and Charlson comorbidity index [28]. Additionally, in the Li et al., the SII was validated as an independent predictor of the composite of all-cause death, non-fatal ischemic stroke, and nonfatal MI in 1701 ACS patients undergoing PCI. Moreover, the addition of SII on top of the GRACE risk score significantly improves the latter one's predictive value [31]. In line with prior studies, we found that a higher SII level remained an independent risk factor of long-term mortality after multivariable adjustment (Table 2). The positive association of SII with impaired LVEF and extensive myocardial necrosis uncovered by our GAM analyses could partially explain the adverse prognostic impact of high SII, given the well-known detrimental effects of decreased LVEF and elevated TnT levels. On the other hand, as an emerging index of prothrombotic activity, a high SII level may indicate the presence of excessive thrombotic burden, which has also been considered an important risk factor of the no-reflow phenomenon and poor survival following AMI [32].

Another interesting finding that we have shown, to our knowledge for the first time, is that the SII was an independent risk factor of poor survival only in patients with diabetes but not in those without diabetes. Our RCS analyses further indicated a linear correlation between SII and long-term mortality in diabetics, which may suggest the clinical utility of anti-inflammatory therapies in this high-risk population. Reduced inflammation response in AMI patients with DM such as treating hyperglycemia will slow down the adverse cardiac remodeling and also reduce the CV outcomes [33]. Emerging evidence has indicated that certain glucose-lowering agents such as metformin and sodium-glucose cotransporter 2 inhibitors could provide survival benefits to patients with AMI partly due to their anti-inflammatory properties [18, 19]. Whether it is possible to prescribe appropriate hypoglycemic therapies based on the SII level to improve the prognosis of AMI patients with diabetes remains to be determined.

Despite the known impacts of leukocytes and platelets on the prognosis of AMI individuals, we found that the significant association between SII and mortality was mainly mediated by neutrophils, particularly in the diabetics (Additional file 1: Table S4). The exact mechanisms cannot be determined in this analysis, while we postulated that the differences in the functional status of neutrophils in diabetics and nondiabetics may be one of the possible explanations. Under diabetic conditions, neutrophils are more prone to producing superoxide and inflammatory cytokines, which results in tissue injuries [34]. On the other hand, the diabetic microenvironment favors the form of neutrophils extracellular traps (NETs) [35]. As shown in the Menegazzo et al., plasma from type 2 DM patients included more NETosis products, such as elastase, oligonucleotides, and double-strand DNA, when compared to nondiabetic individuals [36]. The NETs are pivotal scaffolds in pathologic thrombi and fuel cardiovascular, inflammatory, and thrombotic diseases. It has been well established that NETs are key in promoting DM-related complications [37, 38]. Taken together, our results suggest that a therapeutic strategy targeting neutrophils or NETs may be reasonable in improving the outcomes of AMI patients with DM. Further studies are highly desirable to address this issue.

The present study also has several limitations. First, this was a single-center retrospective cohort study, it was difficult to eliminate underlying selection bias and some unmeasured confounders. Second, due to a lack of data on previous medication usage, such as the use of steroids and antibiotics, which may influence the assessment of SII. However, our study excluded patients with severe inflammation, hematological diseases, or autoimmune diseases, and adjusted for a majority of confounders that may influence blood cell counts, including smoking status, diabetes, and aspirin. Third, as several other inflammatory markers such as monocyte chemoattractant protein-1 (MCP-1) [39] and IL-6 [40] that have been associated with poor CV outcomes in patients with AMI were not available in the NOAFCAMI-SH database, their impacts on the association between SII and clinical outcomes after AMI remained to be elucidated. Fourth, although we excluded patients with severe inflammation and accounted for the effect of CRP that is generally elevated in the setting of acute infection in the multivariable analyses, we still cannot eliminate the impact of acute infection on our results. Finally, we did not investigate the association of dynamic changes in the SII level with long-term mortality, which needs to be further addressed.

## Conclusions

In summary, we demonstrate that high SII is positively associated with myocardial injury and cardiac dysfunction, and is also an independent risk factor for long-term mortality in AMI patients, particularly in diabetics. Given the detrimental prognostic impacts of extensive inflammation response after AMI, further studies are warranted to determine whether the SII could be helpful in the risk stratification of AMI patients with DM as well as in the clinical decision-making of anti-inflammatory therapy utility.

### Supplementary Information

**Additional file 1: Table S1.** Univariable analysis for the all-cause death in the whole cohort. **Table S2.** Baseline characteristics of patients with diabetes by tertiles of systemic-immune inflammation index. **Table S3.** Baseline characteristics of patients without diabetes by tertiles of systemic-immune inflammation index. **Table S4.** Sensitivity analysis: Association between tertiles of the systemic immune-inflammation index and death in a complete dataset (N = 1895). **Table S5.** Sensitivity analysis: Association between tertiles of the systemic immune-inflammation index and death in patients with and without diabetes after further accounting for oral anticoagulants and diuretics. **Table S6.** Sensitivity analysis: Association between tertiles of the systemic immune-inflammation index and death in the overall and diabetic cohorts after further accounting for anti-diabetic agents. **Table S7.** Association between neutrophil counts and clinical outcomes. **Table S8.** Association between lymphocyte counts and clinical outcomes. **Table S9.** Association between platelet counts and clinical outcomes

## Data Availability

The datasets used and/or analyzed during this study are available from the corresponding author on reasonable request.

## Abbreviations

AMI: Acute myocardial infarction
CKD: Chronic kidney disease
DM: Diabetes mellitus
GAM: Generalized additive model
HF: Heart failure
LVEF: Left ventricular ejection fraction
NET: Neutrophils extracellular trap
RCS: Restricted cubic spline
SII: Systemic immune-inflammation index
