# Supplementary material for: Prognostic implications of systemic immune-inflammation index in myocardial infarction patients with and without diabetes: insights from the NOAFCAMI-SH registry
Source: Cardiovasc Diabetol. 2024 Jan 22;23:41. doi: 10.1186/s12933-024-02129-x (PMC10804591; doi:10.1186/s12933-024-02129-x)
Supplement: Supplementary file 1 — Additional file 1: Table S1. Univariable analysis for the all-cause death in the whole cohort. Table S2. Baseline characteristics of patients with diabetes by tertiles of systemic-immune inflammation index. Table S3. Baseline characteristics of patients without diabetes by tertiles of systemic-immune inflammation index. Table S4. Sensitivity analysis: Association between tertiles of the systemic immune-inflammation index and death in a complete dataset (N = 1895). Table S5. Sensitivity analysis: Association between tertiles of the systemic immune-inflammation index and death in patients with and without diabetes after further accounting for oral anticoagulants and diuretics. Table S6. Sensitivity analysis: Association between tertiles of the systemic immune-inflammation index and death in the overall and diabetic cohorts after further accounting for anti-diabetic agents. Table S7. Association between neutrophil counts and clinical outcomes. Table S8. Association between lymphocyte counts and clinical outcomes. Table S9. Association between platelet counts and clinical outcomes [file 12933_2024_2129_MOESM1_ESM.docx]

**Additional file 1**

1. **Supplemental Methods**
2. **Table S1.** Univariable analysis for the all-cause death in the whole cohort
3. **Table S2.** Baseline characteristics of patients with diabetes by tertiles of systemic-immune inflammation index
4. **Table S3.** Baseline characteristics of patients without diabetes by tertiles of systemic-immune inflammation index
5. **Table S4.** Sensitivity analysis: Association between tertiles of the systemic immune-inflammation index and death in a complete dataset (N=1895).
6. **Table S5.** Sensitivity analysis: Association between tertiles of the systemic immune-inflammation index and death in patients with and without diabetes after further accounting for oral anticoagulants and diuretics
7. **Table S6.** Sensitivity analysis: Association between tertiles of the systemic immune-inflammation index and death in the overall and diabetic cohorts after further accounting for anti-diabetic agents
8. **Table S7.** Association between neutrophil counts and clinical outcomes.
9. **Table S8.** Association between lymphocyte counts and clinical outcomes
10. **Table S9.** Association between platelet counts and clinical outcomes

**Supplemental Methods**

**Multiple imputation**

We used multiple imputation methods with chained equations to impute data for all variables with missing information to account for the missing data and protect against the biases due to informative missing data mechanisms. *Age, sex, current smoker, medical history (hypertension, diabetes, dyslipidemia, heart failure, ischemic stroke/TIA, and myocardial infarction), STEMI, heart rate and systolic blood pressure at admission, primary PCI, peak TnT, medications (aspirin, β-blocker, statins, ACEI/ARB/ARNI), as well as study outcomes,* were registered as complete variables in the imputation models, which were applied to generate 20 complete datasets on which we performed the analyses (imputed variables included *C-reactive protein, serum creatinine, and left ventricular ejection fraction*).

**Table S1.** Univariable analysis for the all-cause death in the whole cohort

| **Variable** | **N (%)** | **HR (95%CI)** | **P-value** | **Missing, N** |
| --- | --- | --- | --- | --- |
| Log-transformed SII | - | 2.83 (1.83-4.37) | <0.001 | 0 |
| SII (Tertile 3 vs. Tertiles 1-2) | - | 1.64 (1.25-2.16) | <0.001 | 0 |
| **Demographics** |  |  |  |  |
| Age, yrs | - | 1.09 (1.08-1.10) | <0.001 | 0 |
| Female sex | - | 1.57 (1.17-2.10) | 0.003 | 0 |
| Current smoker | 953 (45.1) | 0.45 (0.34-0.61) | <0.001 | 0 |
| **Comorbidities** |  |  |  |  |
| Hypertension | 1342 (63.6) | 1.38 (1.03-1.86) | 0.032 | 0 |
| Diabetes mellitus | 789 (37.4) | 1.50 (1.14-1.97) | 0.004 | 0 |
| Dyslipidemia | 565 (26.8) | 1.37 (1.03-1.83) | 0.030 | 0 |
| Chronic kidney disease | 165 (7.8) | 4.14 (2.98-5.74) | <0.001 | 0 |
| History of HF | 105 (5.0) | 8.25 (6.00-11.34) | <0.001 | 0 |
| History of MI | 137 (6.5) | 1.77 (1.15-2.73) | 0.010 | 0 |
| History of PCI | 187 (8.9) | 1.84 (1.26-2.69) | 0.002 | 0 |
| History of stroke/TIA | 236 (11.2) | 2.69 (1.96-3.70) | <0.001 | 0 |
| **Admission presentation** |  |  |  |  |
| Out-of-hospital cardiac arrest | 40 (1.9) | 1.22 (0.50-2.95) | 0.667 | 0 |
| STEMI | 1272 (60.3) | 0.54 (0.41-0.71) | <0.001 | 0 |
| Primary PCI | 1778 (84.2) | 0.26 (0.20-0.34) | <0.001 | 0 |
| Systolic BP, +10 mmHg | - | 1.05 (1.00-1.12) | 0.048 | 0 |
| Heart rate, +10 bpm | - | 1.30 (1.21-1.39) | <0.001 | 0 |
| Killip class>I | 279 (13.2) | 4.01 (3.01-5.34) | <0.001 | 0 |
| LVEF, % | - | 0.94 (0.93-0.96) | <0.001 | 97 |
| **Laboratory indices** |  |  |  |  |
| C-reactive protein, mg/L | - | 1.08 (1.05-1.12) | <0.001 | 102 |
| Peak TnT, ng/mL | - | 0.96 (0.93-1.00) | 0.053 | 0 |
| Serum creatinine, mg/dL | - | 4.01 (3.21-5.00) | <0.001 | 26 |
| **Medication at discharge** |  |  |  |  |
| ACEI/ARB/ARNI | 1262 (59.8) | 0.99 (0.75-1.30) | 0.917 | 0 |
| β-blocker | 1528 (72.4) | 0.98 (0.73-1.31) | 0.884 | 0 |
| Aspirin | 1946 (92.2) | 0.51 (0.35-0.74) | <0.001 | 0 |
| Statin | 2026 (96.0) | 0.57 (0.33-0.99) | 0.045 | 0 |

AMI, acute myocardial infarction; ACEI, angiotensin-converting enzyme inhibitor; ARB, angiotensin receptor blocker; ARNI, angiotensin receptor-neprilysin inhibitor; BP, blood pressure; CI, confidence interval; GRACE, Global Registry of Acute Coronary Events; HF, heart failure; HR, hazard ratio; LVEF, left ventricular ejection fraction; MI, myocardial infarction; PCI, percutaneous coronary intervention; SII, systemic immune-inflammation index; TIA, transient ischemic attack.

**Table S2.** Baseline characteristics of patients with diabetes by tertiles of systemic-immune inflammation index

| **Clinical characteristics** | **Diabetic cohort**  **(N=789)** | **Tertiles of systemic-immune inflammation index** | | |  |
| --- | --- | --- | --- | --- | --- |
|  |  | **Tertile 1 (N=277)** | **Tertile 2 (N=258)** | **Tertile 3 (N=254)** | **P-value** |
|  |  | **<613.57** | **613.57~1136.56** | **≥1136.56** |  |
| Age, years | 66.2±12.1 | 66.1±12.0 | 66.2±12.5 | 66.3±12.0 | 0.990 |
| Male sex | 582 (73.8) | 200 (72.2) | 194 (75.2) | 188 (74.0) | 0.730 |
| Current smoker | 331 (42.0) | 113 (40.8) | 116 (45.0) | 102 (40.2) | 0.485 |
| **Comorbidities** |  |  |  |  |  |
| Hypertension | 556 (70.5) | 192 (69.3) | 176 (68.2) | 188 (74.0) | 0.310 |
| Dyslipidemia | 238 (30.2) | 76 (27.4) | 81 (31.4) | 81 (31.9) | 0.463 |
| Chronic kidney disease | 87 (11.0) | 23 (8.3) | 29 (11.2) | 35 (13.8) | 0.132 |
| History of HF | 51 (6.5) | 13 (4.7) | 18 (7.0) | 20 (7.9) | 0.283 |
| History of MI | 65 (8.2) | 28 (10.1) | 19 (7.4) | 18 (7.1) | 0.402 |
| History of PCI | 87 (11.0) | 45 (16.2) | 21 (8.3) | 21 (8.3) | **0.003** |
| History of stroke/TIA | 106 (13.4) | 30 (10.8) | 37 (14.3) | 39 (15.4) | 0.266 |
| **Admission presentation** |  |  |  |  |  |
| STEMI | 446 (56.5) | 118 (42.6) | 154 (59.7) | 174 (68.5) | **<0.001** |
| Anterior location^a^ | 227 (50.9) | 49 (41.5) | 82 (53.2) | 96 (55.2) | 0.181 |
| Systolic BP, mmHg | 141.1±24.4 | 141.9±22.7 | 141.6±23.7 | 139.6±26.8 | 0.507 |
| Heart rate, bpm | 81.8±17.3 | 77.8±15.1 | 81.4±16.0 | 86.7±19.4 | **<0.001** |
| Killip class>I | 105 (13.3) | 28 (10.1) | 29 (11.2) | 48 (18.9) | **0.006** |
| **Laboratory indices** |  |  |  |  |  |
| C-reactive protein, mg/L | 7.00 (3.27-22.00) | 4.21 (3.17-13.90) | 9.17 (3.30-26.50) | 9.70 (3.30-37.20) | **<0.001** |
| Peak TnT, ng/mL | 2.55 (0.63-7.73) | 1.24 (0.44-3.82) | 2.77 (0.95-7.30) | 5.22 (1.28-10.00) | **<0.001** |
| Serum creatinine, mg/dL | 0.98±0.39 | 0.92±0.31 | 0.98±0.40 | 1.03±0.43 | **0.004** |
| Leukocyte count, 10^9^/L | 9.58±3.07 | 7.80±1.95 | 9.25±2.48 | 11.80±3.20 | **<0.001** |
| Neutrophil, count 10^9^/L | 7.15±2.97 | 4.89±1.34 | 6.84±1.95 | 9.94±2.85 | **<0.001** |
| Lymphocyte count, 10^9^/L | 1.74±0.81 | 2.24±0.91 | 1.70±0.58 | 1.23±0.50 | **<0.001** |
| Platelet count, 10^9^/L | 202.4±57.8 | 178.7±48.9 | 202.6±49.8 | 227.9±63.4 | **<0.001** |
| Log-transformed SII | 2.91±0.31 | 2.59±0.15 | 2.91±0.07 | 3.27±0.17 | **<0.001** |
| **Angiographic data** |  |  |  |  |  |
| Primary PCI | 661 (83.8) | 218 (78.7) | 225 (87.2) | 218 (85.8) | **0.016** |
| Infarct-related artery^b^ |  |  |  |  | **0.048** |
| Left anterior descending | 212 (49.9) | 41 (37.6) | 79 (53.4) | 92 (54.8) |  |
| Right coronary artery | 170 (40.0) | 56 (51.4) | 53 (35.8) | 61 (36.3) |  |
| Left circumflex | 43 (10.1) | 12 (11.0) | 16 (10.8) | 15 (8.9) |  |
| **Echocardiographic data** |  |  |  |  |  |
| LAD, mm | 38.5±4.6 | 38.5±4.3 | 38.5±4.8 | 38.4±4.8 | 0.969 |
| LVESD, mm | 31.4±5.5 | 31.0±4.9 | 31.3±5.9 | 31.8±5.7 | 0.330 |
| LVEDD, mm | 45.8±4.8 | 45.7±4.3 | 45.9±5.2 | 45.7±4.8 | 0.796 |
| LVEF, % | 49.5±11.0 | 52.1±10.1 | 49.4±10.6 | 46.7±11.6 | **<0.001** |
| **Medication at discharge** |  |  |  |  |  |
| Aspirin | 722 (91.5) | 248 (89.5) | 239 (92.6) | 235 (92.5) | 0.360 |
| ACEI/ARB/ARNI | 509 (64.5) | 194 (70.0) | 163 (63.2) | 152 (59.8) | **0.043** |
| β-blocker | 587 (74.4) | 203 (73.3) | 187 (72.5) | 197 (77.6) | 0.356 |
| Statin | 755 (95.7) | 265 (95.7) | 248 (96.1) | 242 (95.3) | 0.861 |
| Oral anticoagulants | 4 (0.5) | 3 (1.1) | 0 (0) | 1 (0.4) | 0.276 |
| Diuretics | 148 (18.8) | 41 (14.8) | 46 (17.8) | 61 (24.0) | 0.024 |
| Antidiabetic agents | 636 (80.6) | 220 (79.4) | 205 (79.5) | 211 (83.1) | 0.476 |
| Insulin | 255 (32.3) | 68 (24.6) | 84 (32.6) | 103 (40.6) | <0.001 |
| Metformin | 231 (29.3) | 88 (31.8) | 66 (25.6) | 77 (30.3) | 0.260 |
| Sulfonylureas | 213 (27.0) | 83 (30.0) | 71 (27.5) | 59 (23.2) | 0.214 |
| Glinides | 41 (5.2) | 17 (6.1) | 11 (4.3) | 13 (5.1) | 0.616 |
| Thiazolidinediones | 19 (2.4) | 9 (3.3) | 6 (2.3) | 4 (1.6) | 0.475 |

^a^ For patients with STEMI; ^b^ For patients with STEMI undergoing angiography.

LAD, left atrial diameter; LVEDD, left ventricular end-diastolic diameter; LVEF, left ventricular ejection fraction; LVESD, left ventricular end-systolic diameter; STEMI, ST-segment elevation myocardial infarction. Other abbreviations refer to **Table S1**.

**Table S3.** Baseline characteristics of patients without diabetes by tertiles of systemic-immune inflammation index

| **Clinical characteristics** | **Nondiabetic cohort**  **(N=1322)** | **Tertiles of systemic-immune inflammation index** | | |  |
| --- | --- | --- | --- | --- | --- |
|  |  | **Tertile 1 (N=425)** | **Tertile 2 (N=448)** | **Tertile 3 (N=449)** | **P-value** |
|  |  | **<613.57** | **613.57~1136.56** | **≥1136.56** |  |
| Age, years | 64.6±12.2 | 64.9±12.0 | 63.7±12.3 | 65.1±12.4 | 0.187 |
| Male sex | 1055 (79.8) | 334 (78.6) | 358 (79.9) | 363 (80.8) | 0.706 |
| Current smoker | 622 (47.0) | 190 (44.7) | 227 (50.7) | 205 (45.7) | 0.162 |
| **Comorbidities** |  |  |  |  |  |
| Hypertension | 786 (59.5) | 249 (58.6) | 268 (59.8) | 269 (59.9) | 0.907 |
| Dyslipidemia | 327 (24.7) | 107 (25.2) | 106 (23.7) | 114 (25.4) | 0.808 |
| Chronic kidney disease | 78 (5.9) | 17 (4.0) | 32 (7.1) | 29 (6.5) | 0.119 |
| History of HF | 54 (4.1) | 15 (3.5) | 15 (3.3) | 24 (5.3) | 0.250 |
| History of MI | 72 (5.5) | 29 (6.8) | 22 (4.9) | 21 (4.7) | 0.312 |
| History of PCI | 100 (7.6) | 39 (9.2) | 28 (6.2) | 33 (7.3) | 0.264 |
| History of stroke/TIA | 130 (9.8) | 34 (8.0) | 50 (11.2) | 46 (10.2) | 0.274 |
| **Admission presentation** |  |  |  |  |  |
| STEMI | 826 (62.5) | 209 (49.2) | 285 (63.6) | 332 (73.9) | **<0.001** |
| Anterior location^a^ | 427 (51.7) | 106 (50.7) | 152 (53.3) | 169 (50.9) | 0.557 |
| Systolic BP, mmHg | 136.9±23.4 | 137.4±22.5 | 137.8±23.1 | 135.4±24.5 | 0.267 |
| Heart rate, bpm | 78.3±16.3 | 75.3±16.0 | 78.6±15.0 | 80.9±17.2 | **<0.001** |
| Killip class>I | 174 (13.2) | 40 (9.4) | 47 (10.5) | 87 (19.4) | **<0.001** |
| **Laboratory indices** |  |  |  |  |  |
| C-reactive protein, mg/L | 4.46 (3.02-15.20) | 3.80 (3.02-11.66) | 4.20 (3.17-14.50) | 5.56 (3.02-20.52) | **0.006** |
| Peak TnT, ng/mL | 3.25 (0.92-8.09) | 1.76 (0.43-5.50) | 3.00 (0.97-7.12) | 5.39 (1.92-10.00) | **<0.001** |
| Serum creatinine, mg/dL | 0.94±0.29 | 0.93±0.31 | 0.93±0.31 | 0.94±0.26 | 0.728 |
| Leukocyte count, 10^9^/L | 9.52±3.03 | 7.77±2.16 | 9.33±2.58 | 11.40±3.09 | **<0.001** |
| Neutrophil, count 10^9^/L | 7.13±2.89 | 4.87±1.52 | 6.90±2.03 | 9.51±2.79 | **<0.001** |
| Lymphocyte count, 10^9^/L | 1.71±0.79 | 2.20±0.86 | 1.74±0.64 | 1.21±0.49 | **<0.001** |
| Platelet count, 10^9^/L | 210.0±60.5 | 183.3±48.6 | 209.1±57.4 | 236.1±62.7 | **<0.001** |
| Log-transformed SII | 2.93±0.31 | 2.60±0.15 | 2.91±0.08 | 3.27±0.18 | **<0.001** |
| **Angiographic data** |  |  |  |  |  |
| Primary PCI | 1117 (84.5) | 349 (82.1) | 384 (85.7) | 384 (85.5) | 0.259 |
| Infarct-related artery^b^ |  |  |  |  | 0.145 |
| Left anterior descending | 414 (52.5) | 105 (52.2) | 153 (55.4) | 156 (50.2) |  |
| Right coronary artery | 285 (36.2) | 71 (35.3) | 87 (31.5) | 127 (40.8) |  |
| Left circumflex | 89 (11.3) | 25 (12.4) | 36 (13.0) | 28 (9.0) |  |
| **Echocardiographic data** |  |  |  |  |  |
| LAD, mm | 37.9±4.6 | 38.2±4.5 | 38.0±4.6 | 37.5±4.6 | 0.110 |
| LVESD, mm | 30.7±5.4 | 30.5±5.7 | 30.3±4.8 | 31.3±5.6 | **0.023** |
| LVEDD, mm | 45.5±4.8 | 45.6±5.0 | 45.3±4.2 | 45.6±5.0 | 0.558 |
| LVEF, % | 51.1±10.2 | 53.0±9.6 | 52.1±9.8 | 48.2±10.6 | **<0.001** |
| **Medication at discharge** |  |  |  |  |  |
| Aspirin | 1224 (92.6) | 385 (90.6) | 423 (94.4) | 416 (92.7) | 0.097 |
| ACEI/ARB/ARNI | 753 (57.0) | 251 (59.1) | 266 (59.4) | 236 (52.6) | 0.068 |
| β-blocker | 941 (71.2) | 282 (66.4) | 324 (72.3) | 335 (74.6) | **0.021** |
| Statin | 1271 (96.1) | 412 (96.9) | 431 (96.2) | 428 (95.3) | 0.461 |
| Oral anticoagulants | 8 (0.6) | 3 (0.7) | 2 (0.5) | 3 (0.7) | 0.911 |
| Diuretics | 154 (11.7) | 38 (8.9) | 56 (12.5) | 60 (13.4) | 0.091 |

^a^ For patients with STEMI; ^b^ For patients with STEMI undergoing angiography.

Abbreviations refer to **Tables S1 and S2**.

**Table S4.** Sensitivity analysis: Association between tertiles of the systemic immune-inflammation index and death in a complete dataset (N=1895)

| **Clinical outcomes** | **Log-transformed SII** | **Tertiles of systemic immune-inflammation index** | | | **P for trend** |
| --- | --- | --- | --- | --- | --- |
|  |  | **Tertile 1 (N=618)** | **Tertile 2 (N=630)** | **Tertile 3 (N=647)** |  |
|  |  | **<613.57** | **613.57~1136.56** | **≥1136.56** |  |
| **All-cause death** |  |  |  |  |  |
| **Overall cohort (N=1895)** |  |  |  |  |  |
| Event | - | 52 | 57 | 83 | - |
| Incidence rate (95%CI)^a^ | - | 3.14 (2.39-4.12) | 3.46 (2.67-4.49) | 5.28 (4.25-6.54) | - |
| Unadjusted Model | 2.75 (1.75-4.33)^**^ | Ref. | 1.10 (0.76-1.60) | 1.67 (1.18-2.36)^*^ | 0.003 |
| Adjusted Model 1^b^ | 2.11 (1.40-3.18)^**^ | Ref. | 1.18 (0.81-1.72) | 1.68 (1.18-2.37)^*^ | 0.003 |
| Adjusted Model 2^c^ | 1.59 (1.02-2.50)^*^ | Ref. | 1.08 (0.73-1.59) | 1.53 (1.05-2.24)^*^ | **0.021** |
| **Diabetic cohort (N=700)** |  |  |  |  |  |
| Event | - | 22 | 27 | 38 | - |
| Incidence rate (95%CI)^a^ | - | 3.42 (2.25-5.20) | 4.73 (3.24-6.89) | 7.41 (5.40-10.19) | - |
| Unadjusted Model | 5.03 (2.50-10.15)^**^ | Ref. | 1.38 (0.79-2.42) | 2.13 (1.26-3.60)^*^ | 0.004 |
| Adjusted Model 1^b^ | 4.34 (2.18-8.63)^**^ | Ref. | 1.36 (0.78-2.39) | 2.14 (1.26-3.63)^*^ | 0.004 |
| Adjusted Model 2^c^ | 3.45 (1.64-7.27)^**^ | Ref. | 1.50 (0.83-2.72) | 2.28 (1.25-4.17)^*^ | **0.007** |
| **Nondiabetic cohort (N=1195)** |  |  |  |  |  |
| Event | - | 30 | 30 | 45 | - |
| Incidence rate (95%CI)^a^ | - | 2.96 (2.07-4.24) | 2.79 (1.95-3.99) | 4.24 (3.17-5.68) | - |
| Unadjusted Model | 1.92 (1.04-3.55)^*^ | Ref. | 0.95 (0.57-1.58) | 1.43 (0.90-2.27) | 0.108 |
| Adjusted Model 1^b^ | 1.47 (0.86-2.51) | Ref. | 1.07 (0.64-1.77) | 1.43 (0.90-2.27) | 0.119 |
| Adjusted Model 2^c^ | 0.84 (0.47-1.51) | Ref. | 0.87 (0.51-1.49) | 1.07 (0.65-1.78) | 0.719 |
| **Cardiovascular death** |  |  |  |  |  |
| **Overall cohort (N=1895)** |  |  |  |  |  |
| Event | - | 34 | 38 | 69 | - |
| Incidence rate (95%CI)^a^ | - | 2.05 (1.47-2.87) | 2.31 (1.68-3.17) | 4.39 (3.46-5.55) | - |
| Unadjusted Model | 3.85 (2.29-6.48)^**^ | Ref. | 1.12 (0.70-1.78) | 2.10 (1.39-3.17)^**^ | <0.001 |
| Adjusted Model 1^b^ | 2.88 (1.80-4.62)^**^ | Ref. | 1.19 (0.75-1.90) | 2.12 (1.41-3.20)^**^ | <0.001 |
| Adjusted Model 2^c^ | 1.84 (1.10-3.09)^*^ | Ref. | 1.08 (0.67-1.75) | 1.82 (1.16-2.84)^*^ | **0.005** |
| **Diabetic cohort (N=700)** |  |  |  |  |  |
| Event | - | 16 | 18 | 32 | - |
| Incidence rate (95%CI)^a^ | - | 2.49 (1.52-4.06) | 3.15 (1.99-5.00) | 6.24 (4.42-8.83) | - |
| Unadjusted Model | 6.48 (2.91-14.46)^**^ | Ref. | 1.25 (0.64-2.45) | 2.41 (1.32-4.40)^*^ | 0.003 |
| Adjusted Model 1^b^ | 5.64 (2.57-12.38)^**^ | Ref. | 1.24 (0.63-2.44) | 2.44 (1.34-4.46)^*^ | 0.003 |
| Adjusted Model 2^c^ | 3.55 (1.51-8.35)^*^ | Ref. | 1.39 (0.68-2.82) | 2.34 (1.18-4.63)^*^ | **0.013** |
| **Nondiabetic cohort (N=1195)** |  |  |  |  |  |
| Event | - | 18 | 20 | 37 | - |
| Incidence rate (95%CI)^a^ | - | 1.78 (1.12-2.82) | 1.86 (1.20-2.88) | 3.49 (2.53-4.81) | - |
| Unadjusted Model | 2.86 (1.41-5.84)^*^ | Ref. | 1.05 (0.56-1.99) | 1.96 (1.11-3.44)^*^ | 0.012 |
| Adjusted Model 1^b^ | 2.04 (1.10-3.81)^*^ | Ref. | 1.18 (0.62-2.23) | 1.96 (1.11-3.44)^*^ | 0.015 |
| Adjusted Model 2^c^ | 0.99 (0.50-1.95) | Ref. | 0.98 (0.50-1.90) | 1.40 (0.76-2.59) | 0.231 |

^*^P<0.05; ^**^P<0.001

^a^ Incidence rate was calculated using the total number of deaths during the observational period divided by person-years at risk

^b^ Model 1 included age and sex.

^c^ Model 2 included age, sex, current smoker, comorbidities (hypertension, diabetes, dyslipidemia, CKD, HF, and MI), STEMI, Killip>I, primary PCI, peak TnT, CRP, serum creatinine, LVEF, as well as medications (aspirin, ACEI/ARB/ARNI, β-blocker).

Abbreviations refer to **Tables S1 and S2**.

**Table S5. Sensitivity analysis:** Association between tertiles of systemic immune-inflammation index and death in patients with and without diabetes after further accounting for oral anticoagulants and diuretics

| **Clinical outcomes** | **Log-transformed SII** | **Tertiles of systemic immune-inflammation index** | | | **P for trend** |
| --- | --- | --- | --- | --- | --- |
|  |  | **Tertile 1 (N=702)** | **Tertile 2 (N=706)** | **Tertile 3 (N=703)** |  |
|  |  | **<613.57** | **613.57~1136.56** | **≥1136.56** |  |
| **All-cause death** |  |  |  |  |  |
| **Overall cohort (N=2111)** |  |  |  |  |  |
| Event | - | 57 | 63 | 90 | - |
| Incidence rate (95%CI)^a^ | - | 2.95 (2.28-3.83) | 3.35 (2.62-4.29) | 5.23 (4.25-6.43) | - |
| Unadjusted Model | 2.83 (1.83-4.38)^**^ | Ref. | 1.14 (0.80-1.63) | 1.75 (1.26-2.44)^**^ | <0.001 |
| Adjusted Model^b^ | 1.57 (1.02-2.44)^*^ | Ref. | 1.02 (0.71-1.48) | 1.53 (1.07-2.20)^*^ | 0.017 |
| **Diabetic cohort (N=789)** |  |  |  |  |  |
| Event | - | 25 | 32 | 39 | - |
| Incidence rate (95%CI)^a^ | - | 3.32 (2.24-4.91) | 4.75 (3.36-6.72) | 6.97 (5.09-9.54) | - |
| Unadjusted Model | 4.47 (2.28-8.78)^**^ | Ref. | 1.43 (0.85-2.41) | 2.05 (1.24-3.40)^*^ | 0.005 |
| Adjusted Model^b^ | 3.05 (1.47-6.35)^*^ | Ref. | 1.32 (0.76-2.29) | 2.01 (1.13-3.58)^*^ | 0.019 |
| **Nondiabetic cohort (N=1322)** |  |  |  |  |  |
| Event | - | 32 | 31 | 51 | - |
| Incidence rate (95%CI)^a^ | - | 2.72 (1.92-3.84) | 2.57 (1.81-3.66) | 4.39 (3.34-5.77) | - |
| Unadjusted Model | 2.23 (1.24-4.01)^*^ | Ref. | 0.95 (0.58-1.56) | 1.61 (1.03-2.51)^*^ | 0.026 |
| Adjusted Model^b^ | 0.97 (0.56-1.70)^*^ | Ref. | 0.85 (0.51-0.86) | 1.20 (0.74-1.96) | 0.380 |
| **Cardiovascular death** |  |  |  |  |  |
| **Overall cohort (N=2111)** |  |  |  |  |  |
| Event | - | 38 | 41 | 75 | - |
| Incidence rate (95%CI)^a^ | - | 1.97 (1.43-2.70) | 2.18 (1.61-2.96) | 4.36 (3.47-5.46) | - |
| Unadjusted Model | 3.88 (2.35-6.39)^**^ | Ref. | 1.10 (0.71-1.72) | 2.17 (1.46-3.20)^**^ | <0.001 |
| Adjusted Model^b^ | 1.86 (1.12-3.06)^*^ | Ref. | 1.01 (0.64-1.60) | 1.82 (1.19-2.79)^*^ | 0.004 |
| **Diabetic cohort (N=789)** |  |  |  |  |  |
| Event | - | 19 | 20 | 33 | - |
| Incidence rate (95%CI)^a^ | - | 2.52 (1.61-3.95) | 2.97 (1.92-4.60) | 5.90 (4.19-8.29) | - |
| Unadjusted Model | 5.61 (2.58-12.17)^**^ | Ref. | 1.16 (0.62-2.18) | 2.23 (1.27-3.93)^*^ | 0.004 |
| Adjusted Model^b^ | 3.49 (1.50-8.09)^*^ | Ref. | 1.11 (0.57-2.16) | 2.14 (1.12-4.10)^*^ | 0.023 |
| **Nondiabetic cohort (N=1322)** |  |  |  |  |  |
| Event | - | 19 | 21 | 42 | - |
| Incidence rate (95%CI)^a^ | - | 1.61 (1.03-2.53) | 1.74 (1.14-2.67) | 3.61 (2.67-4.89) | - |
| Unadjusted Model | 3.25 (1.65-6.41)^**^ | Ref. | 1.08 (0.58-2.01) | 2.22 (1.29-3.81)^*^ | 0.002 |
| Adjusted Model^b^ | 1.14 (0.60-2.19) | Ref. | 0.98 (0.51-1.89) | 1.58 (0.87-2.87) | 0.096 |

^*^P<0.05; ^**^P<0.001

^a^ Incidence rate was calculated using the total number of deaths during the observational period divided by person-years at risk

^b^ Adjusted Model included age, sex, current smoker, comorbidities (hypertension, diabetes, dyslipidemia, CKD, HF, and MI), STEMI, Killip>I, primary PCI, peak TnT, CRP, serum creatinine, LVEF, as well as medications (aspirin, ACEI/ARB/ARNI, β-blocker, OAC, and diuretics).

CKD, chronic kidney disease; CRP, C-reactive protein; HF, heart failure; LVEF, left ventricular ejection fraction; OAC, oral anticoagulant; PCI, percutaneous coronary intervention; SII, systemic immune-inflammation index.

**Table S6. Sensitivity analysis:** Association between tertiles of systemic immune-inflammation index and death in the overall and diabetic cohorts after further accounting for anti-diabetic agents

| **Clinical outcomes** | **Log-transformed SII** | **Tertiles of systemic immune-inflammation index** | | | **P for trend** |
| --- | --- | --- | --- | --- | --- |
|  |  | **Tertile 1 (N=702)** | **Tertile 2 (N=706)** | **Tertile 3 (N=703)** |  |
|  |  | **<613.57** | **613.57~1136.56** | **≥1136.56** |  |
| **All-cause death** |  |  |  |  |  |
| **Overall cohort (N=2111)** |  |  |  |  |  |
| Event | - | 57 | 63 | 90 | - |
| Incidence rate (95%CI)^a^ | - | 2.95 (2.28-3.83) | 3.35 (2.62-4.29) | 5.23 (4.25-6.43) | - |
| Unadjusted Model | 2.83 (1.83-4.38)^**^ | Ref. | 1.14 (0.80-1.63) | 1.75 (1.26-2.44)^**^ | <0.001 |
| Adjusted Model^b^ | 1.57 (1.02-2.42)^*^ | Ref. | 1.03 (0.71-1.49) | 1.52 (1.06-2.18) | 0.020 |
| **Diabetic cohort (N=789)** |  |  |  |  |  |
| Event | - | 25 | 32 | 39 | - |
| Incidence rate (95%CI)^a^ | - | 3.32 (2.24-4.91) | 4.75 (3.36-6.72) | 6.97 (5.09-9.54) | - |
| Unadjusted Model | 4.47 (2.28-8.78)^**^ | Ref. | 1.43 (0.85-2.41) | 2.05 (1.24-3.40)^*^ | 0.005 |
| Adjusted Model^b^ | 2.82 (1.36-5.84)^*^ | Ref. | 1.27 (0.73-2.19) | 1.92 (1.09-3.41)^*^ | 0.027 |
| **Cardiovascular death** |  |  |  |  |  |
| **Overall cohort (N=2111)** |  |  |  |  |  |
| Event | - | 38 | 41 | 75 | - |
| Incidence rate (95%CI)^a^ | - | 1.97 (1.43-2.70) | 2.18 (1.61-2.96) | 4.36 (3.47-5.46) | - |
| Unadjusted Model | 3.88 (2.35-6.39)^**^ | Ref. | 1.10 (0.71-1.72) | 2.17 (1.46-3.20)^**^ | <0.001 |
| Adjusted Model^b^ | 1.82 (1.11-3.00)^*^ | Ref. | 0.99 (0.63-1.57) | 1.76 (1.15-2.70)^*^ | 0.006 |
| **Diabetic cohort (N=789)** |  |  |  |  |  |
| Event | - | 19 | 20 | 33 | - |
| Incidence rate (95%CI)^a^ | - | 2.52 (1.61-3.95) | 2.97 (1.92-4.60) | 5.90 (4.19-8.29) | - |
| Unadjusted Model | 5.61 (2.58-12.17)^**^ | Ref. | 1.16 (0.62-2.18) | 2.23 (1.27-3.93)^*^ | 0.004 |
| Adjusted Model^b^ | 3.03 (1.31-7.00)^*^ | Ref. | 1.05 (0.54-2.04) | 1.94 (1.02-3.69)^*^ | 0.042 |

^*^P<0.05; ^**^P<0.001

^a^ Incidence rate was calculated using the total number of deaths during the observational period divided by person-years at risk

^b^ Adjusted Model included age, sex, current smoker, comorbidities (hypertension, diabetes, dyslipidemia, CKD, HF, and MI), STEMI, Killip>I, primary PCI, peak TnT, CRP, serum creatinine, LVEF, as well as medications (aspirin, ACEI/ARB/ARNI, β-blocker, and anti-diabetic agents).

CKD, chronic kidney disease; CRP, C-reactive protein; HF, heart failure; LVEF, left ventricular ejection fraction; OAC, oral anticoagulant; PCI, percutaneous coronary intervention; SII, systemic immune-inflammation index.

**Table S7.** Association between neutrophil counts and clinical outcomes

| **Clinical outcomes** | **Log-transformed**  **Neutrophil counts** | **P value** |
| --- | --- | --- |
| **All-cause death** |  |  |
| **Overall cohort (N=2111)** |  |  |
| Unadjusted Model | 1.24 (0.58-2.67) | 0.580 |
| Adjusted Model 1^a^ | 2.97 (1.41-6.24) | 0.004 |
| Adjusted Model 2^b^ | 1.53 (0.69-3.41) | 0.295 |
| **Diabetic cohort (N=789)** |  |  |
| Unadjusted Model | 3.22 (1.02-10.18) | 0.046 |
| Adjusted Model 1^a^ | 5.81 (1.87-18.10) | 0.002 |
| Adjusted Model 2^b^ | 4.59 (1.26-16.70) | **0.024** |
| **Nondiabetic cohort (N=1322)** |  |  |
| Unadjusted Model | 0.61 (0.22-1.70) | 0.346 |
| Adjusted Model 1^a^ | 1.85 (0.69-4.95) | 0.222 |
| Adjusted Model 2^b^ | 0.64 (0.22-1.86) | 0.416 |
| **Cardiovascular death** |  |  |
| **Overall cohort (N=2111)** |  |  |
| Unadjusted Model | 2.01 (0.82-4.95) | 0.129 |
| Adjusted Model 1^a^ | 4.67 (1.96-11.14) | 0.001 |
| Adjusted Model 2^b^ | 2.01 (0.79-5.12) | 0.145 |
| **Diabetic cohort (N=789)** |  |  |
| Unadjusted Model | 3.75 (0.99-14.16) | 0.051 |
| Adjusted Model 1^a^ | 7.03 (1.89-26.12) | 0.004 |
| Adjusted Model 2^b^ | 5.37 (1.22-23.68) | **0.031** |
| **Nondiabetic cohort (N=1322)** |  |  |
| Unadjusted Model | 1.24 (0.36-4.20) | 0.733 |
| Adjusted Model 1^a^ | 3.44 (1.08-11.01) | 0.037 |
| Adjusted Model 2^b^ | 0.95 (0.27-3.28) | 0.930 |

^a^ Model 1 included age and sex.

^b^ Model 2 included age, sex, current smoker, comorbidities (hypertension, diabetes, dyslipidemia, CKD, HF, and MI), STEMI, Killip>I, primary PCI, peak TnT, CRP, serum creatinine, LVEF, as well as medications (aspirin, ACEI/ARB/ARNI, β-blocker).

Abbreviations refer to **Tables S1 and S2**.

**Table S8.** Association between lymphocyte counts and clinical outcomes

| **Clinical outcomes** | **Log-transformed**  **lymphocyte counts** | **P value** |
| --- | --- | --- |
| **All-cause death** |  |  |
| **Overall cohort (N=2111)** |  |  |
| Unadjusted Model | 0.10 (0.06-0.19) | <0.001 |
| Adjusted Model 1^a^ | 0.48 (0.24-0.95) | 0.036 |
| Adjusted Model 2^b^ | 0.74 (0.36-1.49) | 0.393 |
| **Diabetic cohort (N=789)** |  |  |
| Unadjusted Model | 0.10 (0.04-0.29) | <0.001 |
| Adjusted Model 1^a^ | 0.30 (0.10-0.86) | 0.025 |
| Adjusted Model 2^b^ | 0.58 (0.19-1.74) | 0.336 |
| **Nondiabetic cohort (N=1322)** |  |  |
| Unadjusted Model | 0.09 (0.04-0.21) | <0.001 |
| Adjusted Model 1^a^ | 0.62 (0.25-1.56) | 0.313 |
| Adjusted Model 2^b^ | 0.99 (0.39-2.50) | 0.984 |
| **Cardiovascular death** |  |  |
| **Overall cohort (N=2111)** |  |  |
| Unadjusted Model | 0.08 (0.04-1.16) | <0.001 |
| Adjusted Model 1^a^ | 0.29 (0.13-0.64) | 0.002 |
| Adjusted Model 2^b^ | 0.57 (0.25-1.28) | 0.172 |
| **Diabetic cohort (N=789)** |  |  |
| Unadjusted Model | 0.07 (0.02-0.22) | <0.001 |
| Adjusted Model 1^a^ | 0.17 (0.05-0.56) | 0.004 |
| Adjusted Model 2^b^ | 0.42 (0.12-1.48) | 0.182 |
| **Nondiabetic cohort (N=1322)** |  |  |
| Unadjusted Model | 0.07 (0.03-0.19) | <0.001 |
| Adjusted Model 1^a^ | 0.41 (0.14-1.19) | 0.100 |
| Adjusted Model 2^b^ | 0.84 (0.29-2.47) | 0.752 |

^a^ Model 1 included age and sex.

^b^ Model 2 included age, sex, current smoker, comorbidities (hypertension, diabetes, dyslipidemia, CKD, HF, and MI), STEMI, Killip>I, primary PCI, peak TnT, CRP, serum creatinine, LVEF, as well as medications (aspirin, ACEI/ARB/ARNI, β-blocker).

Abbreviations refer to **Tables S1 and S2.**

**Table S9.** Association between platelet counts and clinical outcomes

| **Clinical outcomes** | **Log-transformed**  **platelet counts** | **P value** |
| --- | --- | --- |
| **All-cause death** |  |  |
| **Overall cohort (N=2111)** |  |  |
| Unadjusted Model | 0.75 (0.26-2.20) | 0.604 |
| Adjusted Model 1^a^ | 3.28 (1.17-9.20) | 0.024 |
| Adjusted Model 2^b^ | 3.24 (1.16-9.02) | **0.026** |
| **Diabetic cohort (N=789)** |  |  |
| Unadjusted Model | 1.87 (0.36-9.68) | 0.457 |
| Adjusted Model 1^a^ | 4.64 (0.91-23.61) | 0.064 |
| Adjusted Model 2^b^ | 4.36 (0.94-20.15) | 0.063 |
| **Nondiabetic cohort (N=1322)** |  |  |
| Unadjusted Model | 0.44 (0.11-1.84) | 0.262 |
| Adjusted Model 1^a^ | 2.83 (0.75-10.69) | 0.125 |
| Adjusted Model 2^b^ | 1.91 (0.47-7.71) | 0.366 |
| **Cardiovascular death** |  |  |
| **Overall cohort (N=2111)** |  |  |
| Unadjusted Model | 0.81 (0.23-2.83) | 0.740 |
| Adjusted Model 1^a^ | 3.07 (0.92-10.30) | 0.069 |
| Adjusted Model 2^b^ | 3.23 (0.98-10.69) | 0.057 |
| **Diabetic cohort (N=789)** |  |  |
| Unadjusted Model | 1.66 (0.25-10.99) | 0.599 |
| Adjusted Model 1^a^ | 3.65 (0.56-23.79) | 0.176 |
| Adjusted Model 2^b^ | 3.43 (0.60-19.59) | 0.171 |
| **Nondiabetic cohort (N=1322)** |  |  |
| Unadjusted Model | 0.55 (0.10-2.96) | 0.486 |
| Adjusted Model 1^a^ | 2.99 (0.62-14.55) | 0.174 |
| Adjusted Model 2^b^ | 2.10 (0.40-10.94) | 0.383 |

^a^ Model 1 included age and sex.

^b^ Model 2 included age, sex, current smoker, comorbidities (hypertension, diabetes, dyslipidemia, CKD, HF, and MI), STEMI, Killip>I, primary PCI, peak TnT, CRP, serum creatinine, LVEF, as well as medications (aspirin, ACEI/ARB/ARNI, β-blocker).

Abbreviations refer to **Tables S1 and S2**.
